# The efficacy of continuous versus single-injection femoral nerve block in Total knee Arthroplasty: a systematic review and meta-analysis

**DOI:** 10.1186/s12891-020-3148-1

**Published:** 2020-02-24

**Authors:** Hsuan-Hsiao Ma, Te-Feng Arthur Chou, Shang-Wen Tsai, Cheng-Fong Chen, Po-Kuei Wu, Wei-Ming Chen

**Affiliations:** 1 Department of Orthopaedics and Traumatology, Taipei Veterans General Hospital, No. 201, Sec 2, Shi-Pai Road, Taipei, 112 Taiwan; 2 Department of Orthopaedics, School of Medicine, National Yang-Ming University, Taipei, Taiwan

**Keywords:** Continuous, Femoral nerve block, Nerve block, Pain, Single-injection, Total knee arthroplasty

## Abstract

**Background:**

Continuous femoral nerve block (cFNB) has been developed to extend the analgesic effect since the efficacy of single-injection femoral nerve block (sFNB) is often limited to approximately 16–24 h. The aim of this meta-analysis was to validate the add-on effect of cFNB in the setting of a multimodal analgesic protocol.

**Methods:**

We performed a comprehensive literature review on Web of Science, Embase, the Cochrane Library and PubMed. Eight randomized controlled trials (*N* = 626) that compared the efficacy of cFNB with sFNB were included. The primary outcome domains consist of visual analog scale (VAS) score at postoperative 24 and 48 h. The secondary outcome domains include opioid consumption, length of hospital stay and incidence of nausea.

**Results:**

Our analysis revealed that cFNB was associated with a lower VAS score at 24 h (SMD: -0.277;95% CI − 0.503 to − 0.05). However, the difference of VAS score did not meet the minimal clinically importance difference for total knee arthroplasty (TKA). VAS score at 48 h was similar between the cFNB and sFNB group. The cFNB group was associated with less amount of opioids consumed at both 24(SMD: -1.056;95% CI − 1.737 to − 0.375) and 48 h(SMD: -1.040;95% CI − 1.790 to − 0.289). Length of hospital stay and incidence of nausea were similar between the two groups.

**Conclusion:**

In the setting of a multimodal analgesic protocol, patients might benefit from cFNB with regards to a reduced need of opioids in the early postoperative period. However, we did not find a clinically significant difference in pain scores at different time points between the cFNB and sFNB group.

**Level of evidence:**

I; meta-analysis.

## Background

Postoperative pain management is one of the key components in the enhanced recovery after surgery (ERAS) pathway for total knee arthroplasty (TKA) [1]. To manage postoperative pain and facilitate early mobilization and rehabilitation, the concept of multimodal analgesic protocol has been employed [2–4]. Femoral nerve block is a regional nerve block frequently performed after TKAs as part of a multimodal analgesia regimen [5–11]. Currently, there are two commonly used methods to administer femoral nerve blocks, the continuous femoral nerve block (cFNB) and single-shot femoral nerve block (sFNB). FNB has been proven to have a beneficial effect on pain management but it remains uncertain whether cFNB can lead to additional benefits compared with that of sFNB in the setting of multimodal analgesic protocols (eg. pain scores at different time points, amount of opioid consumption, adverse effects of opioids, length of hospital stay and functional outcomes) [9–14]. Due to the mixed results in current literature, it is undetermined whether the additional benefits of cFNB over sFNB are worthy of its extra cost and time involved. Therefore, the aim of this meta-analysis was to determine the efficacy of cFNB compared with the sFNB group and whether cFNB is a superior postoperative pain management modality in patients who had undergone TKA.

## Methods

### Search strategy

We followed the statement on preferred reporting items for systematic reviews and meta-analysis (PRISMA) guidelines (Table 1) and searched a comprehensive search on databases including PubMed, Embase, the Cochrane Library and Web of Science from the earliest record to September 2019. The following searching strategy was similar to the article we have published before [15]. We reviewed all of the articles that discussed continuous femoral nerve block (cFNB) versus single-injection femoral nerve block (sFNB) in TKA. Prior to analysis, we reviewed all included studies that evaluated cFNB or sFNB for the relief of postoperative pain in patients who had received TKA surgery and excluded studies not written in English or when the full article was unavailable. The keywords were with the following combinations: (femoral nerve block OR regional anesthesia OR continuous femoral nerve block OR nerve block) AND (total knee arthroplasty OR total knee replacement). We included only RCTs and excluded comparative experimental trials, single-armed follow-up studies, case series and case reports. All the included studies comprised at least two arms: cFNB and sFNB.

**Table 1:** Characteristics of included studies

Year/First author	Study design	Enrolled Sample number (G1/G2)	Comparing	Anesthesia	Regimen of first bolus in sFNB/cFNB	Regimen and rate of infusion in cFNB		Outcome measurement					
								a	b	c	d	e	f
2019 Angers	RCT	45/45	cFNB, sFNB	SA/GA	20 ml, 0.05% Ropi	0.15% Ropi, 7 ml/hr						V	
2018 Dixit	RCT	44/41	cFNB, sFNB	SA	20-25 ml, 0.5% Ropi	0.2% Ropi, rate not mentioned		V	V	V	V	V	V
2015 Wyatt	RCT	42/42	cFNB, sFNB	SA/GA	20 ml, 0.25% Bupi	0.125% Bupi 10 ml/hr		V	V			V	
2013 Chan	RCT	65/69	cFNB, sFNB	SA/GA	20 ml, 0.25% Bupi with 1:400,000 Epi	0.125% Bupi 4 ml/hr		V	V	V	V	V	V
2013 Albrecht	RCT	60/33	cFNB, sFNB	SA	sFNB: 30 ml, 0.375% Ropi with 1:400,000 Epi cFNB: 20 ml, 0.2% Ropi with 1:400,000 Epi	Ropi, 10 mg/hr. 10 mg bolus allowed every 30 mins		V	V	V	V		V
2010 Park	RCT	60/20	cFNB, sFNB	SA	20 ml, 0.125% Bupi with 1:200,000 Epi	0.125% Bupi 2,4,6 ml/hr		V	V	V	V		
2006 Salinas	RCT	18/18	cFNB, sFNB	SA	30 ml, 0.375% Ropi with 1:400,000 Epi	0.2% Ropi 10 ml/hr		V	V	V	V	V	
1996 Hirst	RCT	11/11	cFNB, sFNB	GA	20 ml, 0.5% Bupi with 1:200,000 Epi	0.125% Bupi 6 ml/hr		V	V	V	V		V

G1 group: study group (cFNB); G2 Group: control group (sFNB)

Outcome measure: a = VAS at 24 h b = VAS at 48 h, c = amount of analgesics consumed within 24 h, d = amount of analgesics consumed within 48 h, e = Length of hospital stay, f = nausea or vomiting event

*FNB* femoral nerve block; *SA* spinal anesthesia; *GA* general anesthesia

*Bupi* Bupivacaine; *Epi* Epinephrine; *Ropi* Ropivacaine

### Eligibility criteria

The included trials (1)enrolled patients who had received unilateral TKA (2) randomized them to receive intervention of cFNB as the pain control method for TKA or sFNB as the primary method to manage pain (3) compared outcome parameters of visual analog scale (VAS) at postoperative 24 and 48 h, cumulative dose of opioids consumption at postoperative 24 and 48 h, length of hospital stay and incidence of postoperative nausea (4)conducted follow-up rate at least 80% and included 1 of the above outcome parameters.

### Data extraction and quality assessment

A pair of reviewers independently abstracted each identified article. The included data were study, patient characteristics, enrolled sample number, type of treatment arms, anesthesia method, regimen of the first bolus in sFNB/cFNB, regimen and rate of infusion in cFNB. The outcome parameters were pain, cumulative dose of opioids consumption, length of hospital stay and incidence of nausea after the surgery were listed in Table 1.

### Data synthesis and analysis

Continuous outcome measures such as postoperative VAS score at different time, the cumulative dose of opioids consumed at postoperative 24 and 48 h, length of hospital stay were pooled and standardized mean differences (SMDs) were calculated. Negative SMD values indicated that cFNB was a more favorable treatment option. The incidence of nausea after surgery (assessed with odds ratio) were reported as binary outcomes. A random effect model was utilized to pool individual SMDs and ORs. Analyses were performed using Comprehensive Meta-Analysis (CMA) software, version 3 (Biostat, Englewood, NJ, USA). Between-trial heterogeneity was determined by using *I*^*2*^ tests; values > 50% were regarded as considerable heterogeneity. Statistical significance was defined as *p*-values < 0.05. Using the Cochrane tool for assessing risk of bias in randomized trials, a pair of reviewers independently evaluated each included study and documented their potential for selection bias, performance bias, detection bias, attrition bias, and reporting bias using. Funnel plots were constructed to visually detect the presence of publication bias.

## Results

**Fig. 1:**
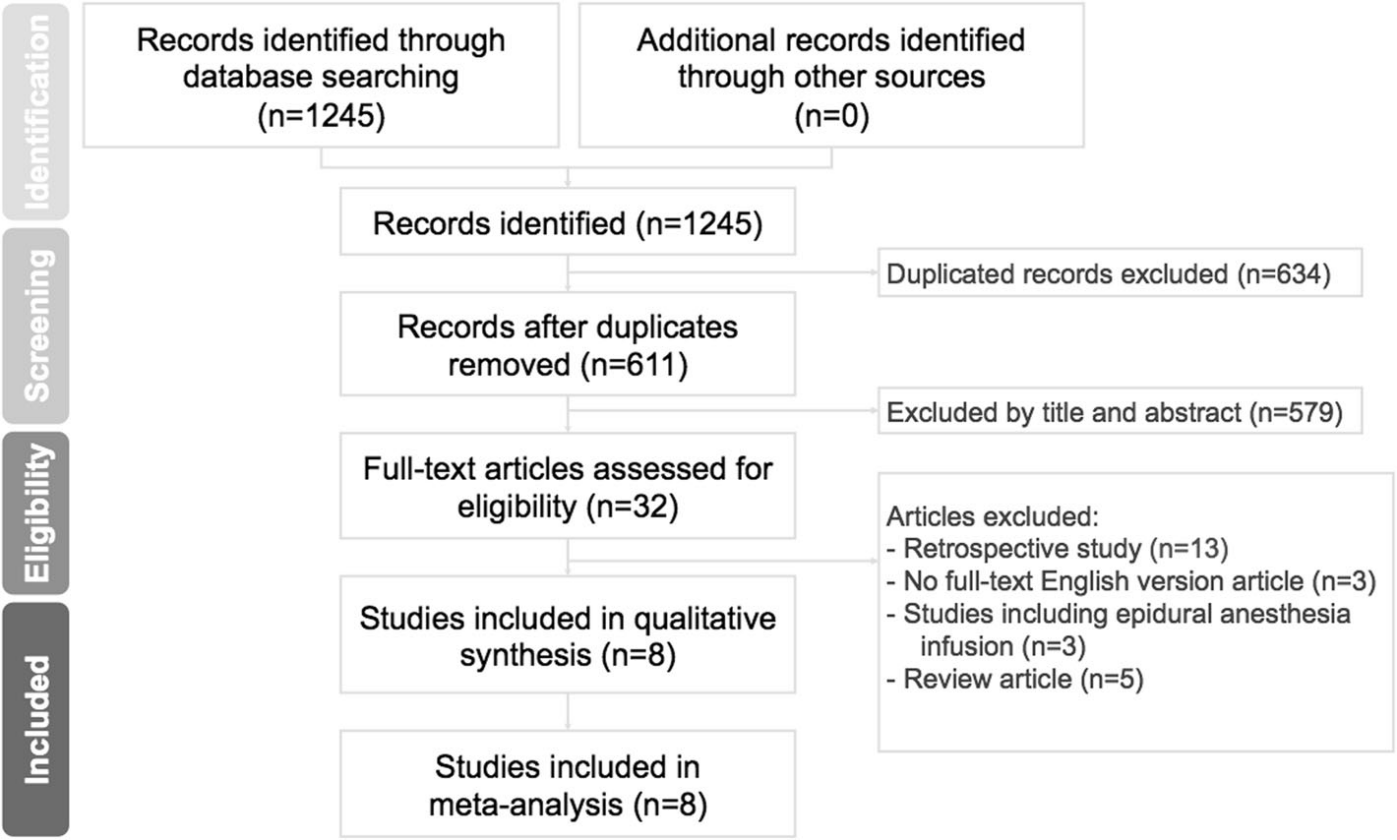
Preferred reporting items for systematic reviews and meta-analysis (PRISMA) study flow diagram

Of 1245 relevant articles identified, 634 duplicate records were removed. We further excluded 579 articles after reading titles and abstracts. Twenty-four studies were excluded after reading the full article, based on the inclusion criteria. There was agreement between reviewers at the full-text review stage and eight RCTs that compared the efficacy of cFNB with sFNB in TKA were included. (Fig. 1) Study details of all 8 studies are listed in Table 1.

### Outcomes for cFNB vs sFNB

#### Pain relief assessed at postoperative 24 hours and 48 h

**Fig. 2:**
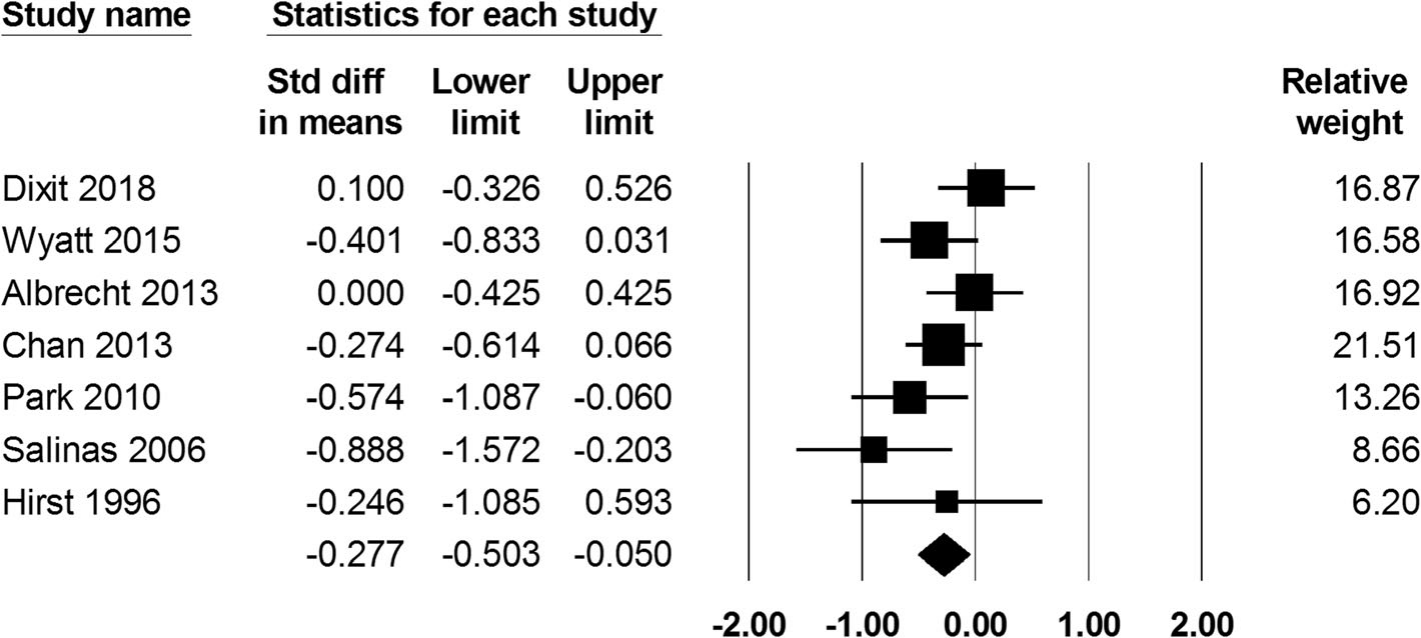
Standardized mean difference (SMD) for VAS score at postoperative 24 h with continuous femoral nerve block (cFNB) versus single-injection femoral nerve block (sFNB)

Of 7 studies (a total of 534 patients), the VAS score at postoperative 24 h. cFNB had lower VAS score at postoperative 24 h comparing with the sFNB group (SMD: -0.277; 95% CI − 0.503 to − 0.05; heterogeneity: I^2^ = 35.181; Fig. 2). VAS score at 24 h in the cFNB and sFNB group were 47.1 mm and 47.6 mm, respectively. The difference in VAS scores between the two groups (0.5 mm) did not reach the minimal clinically important difference (MCID) for TKA, which was reported to be 16.1 mm [16].

**Fig. 3:**
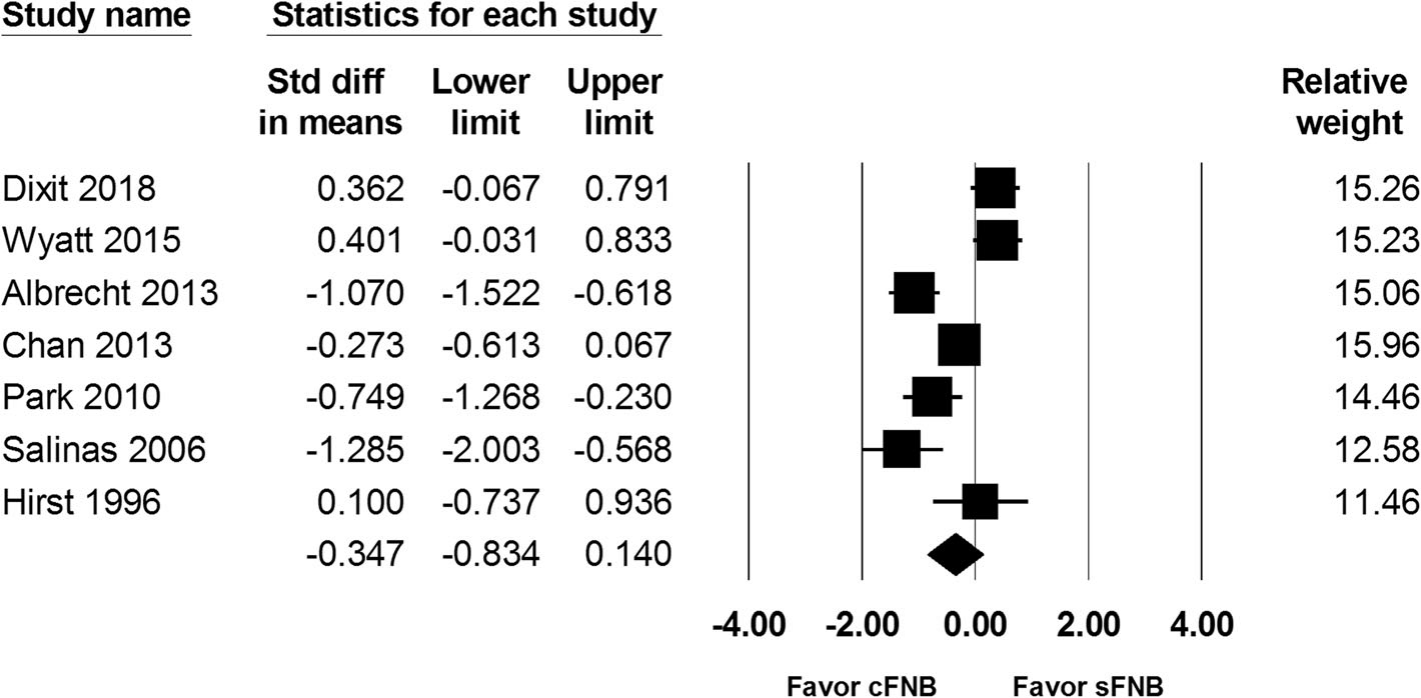
Standardized mean difference (SMD) for VAS score at postoperative 48 h with continuous femoral nerve block (cFNB) versus single-injection femoral nerve block (sFNB)

The VAS score at postoperative 48 h was recorded in 7 studies (a total of 534 patients). VAS score at 48 h in the cFNB and sFNB group were 49.1 mm and 47.8 mm, respectively. The VAS scores at 48 h were similar between the two groups (SMD: -0.347; 95% CI − 0.834 to 0.140; Heterogeneity: I^2^ = 85.49; Fig. 3).

#### Total amount of opioids consumed at postoperative 24 and 48 hours

**Fig. 4:**
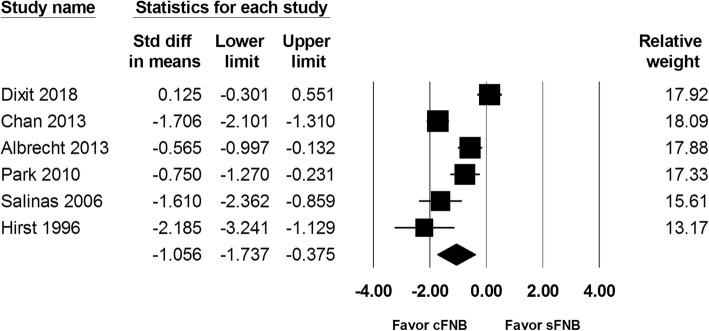
Standardized mean difference (SMD) for total amount of opioids consumed at postopertaive 24 h with continuous femoral nerve block (cFNB) versus single-injection femoral nerve block (sFNB)

**Fig. 5:**
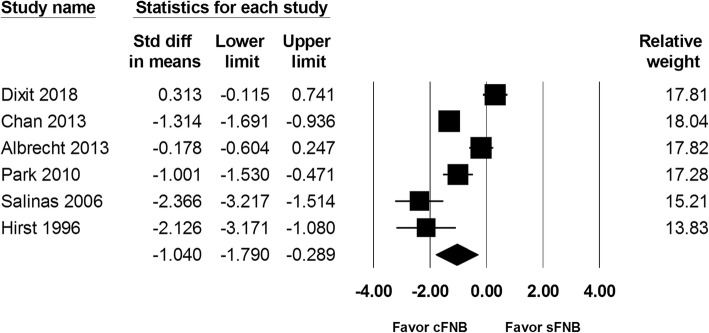
Standardized mean difference (SMD) for total amount of opioids consumed at postopertaive 48 h with continuous femoral nerve block (cFNB) versus single-injection femoral nerve block (sFNB)

Total amount of opioids consumed at postoperative 24 h and 48 h were reported in 6 studies and included 450 and 447 patients, respectively. The total amount of opioids used at postoperative 24 h in the cFNB and sFNB group were 16.6 mg and 25.7 mg, respectively. At postoperative 48 h, a total of 31.4 mg and 42.1 mg of opioids were consumed in the cFNB and sFNB group, respectively. The results showed that cFNB group was associated with a lower opioids consumption at both postoperative 24 h (SMD: -1.056; 95% CI − 1.737 to − 0.375; Heterogeneity: I^2^ = 89.98; Fig. 4) and 48 h (SMD: -1.040; 95% CI − 1.790 to − 0.289; Heterogeneity: I^2^ = 91.73; Fig. 5), compared with the sFNB group.

#### Length of hospital stay

**Fig. 6:**
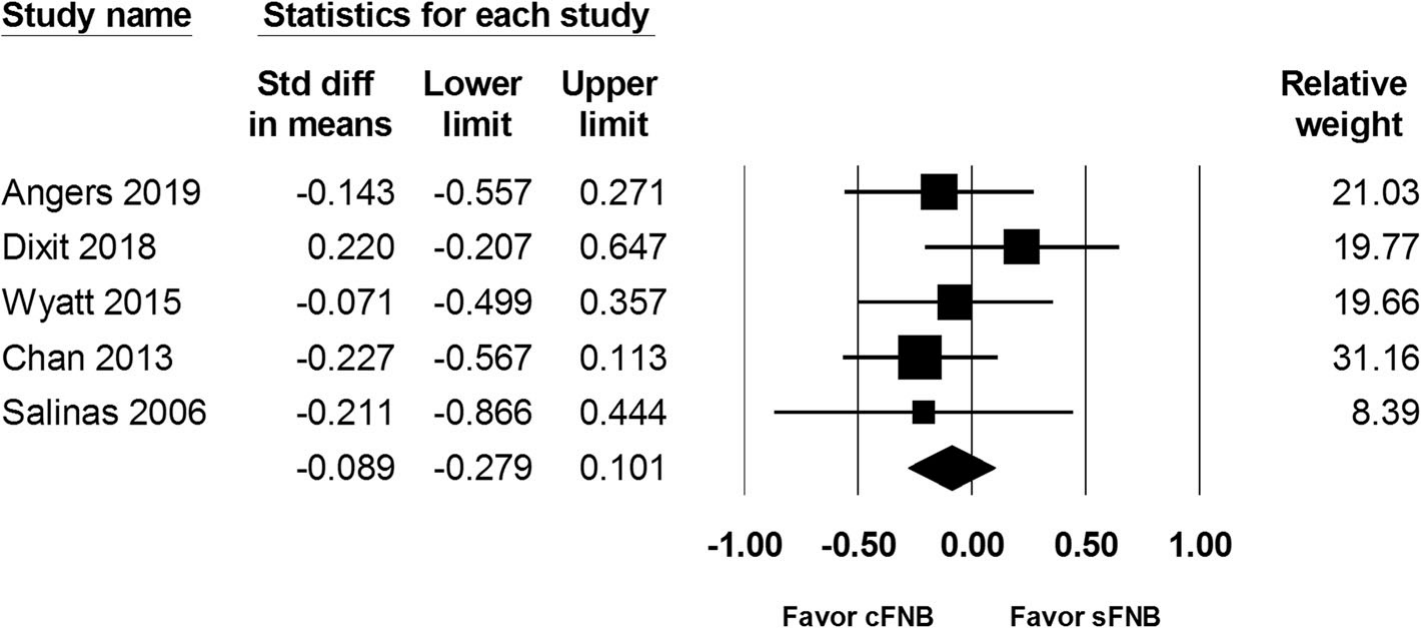
Standardized mean difference (SMD) for length of hospital stay with continuous femoral nerve block (cFNB) versus single-injection femoral nerve block (sFNB)

Of 5 studies (a total of 429 patients), the length of hospital study showed no significant difference found between the cFNB and the sFNB group (SMD: -0.089; 95% CI − 0.279 to 0.101; Heterogeneity: I^2^ = 0.00; Fig. 6).

*Incidence of Postoperative Nausea.*

**Fig. 7:**
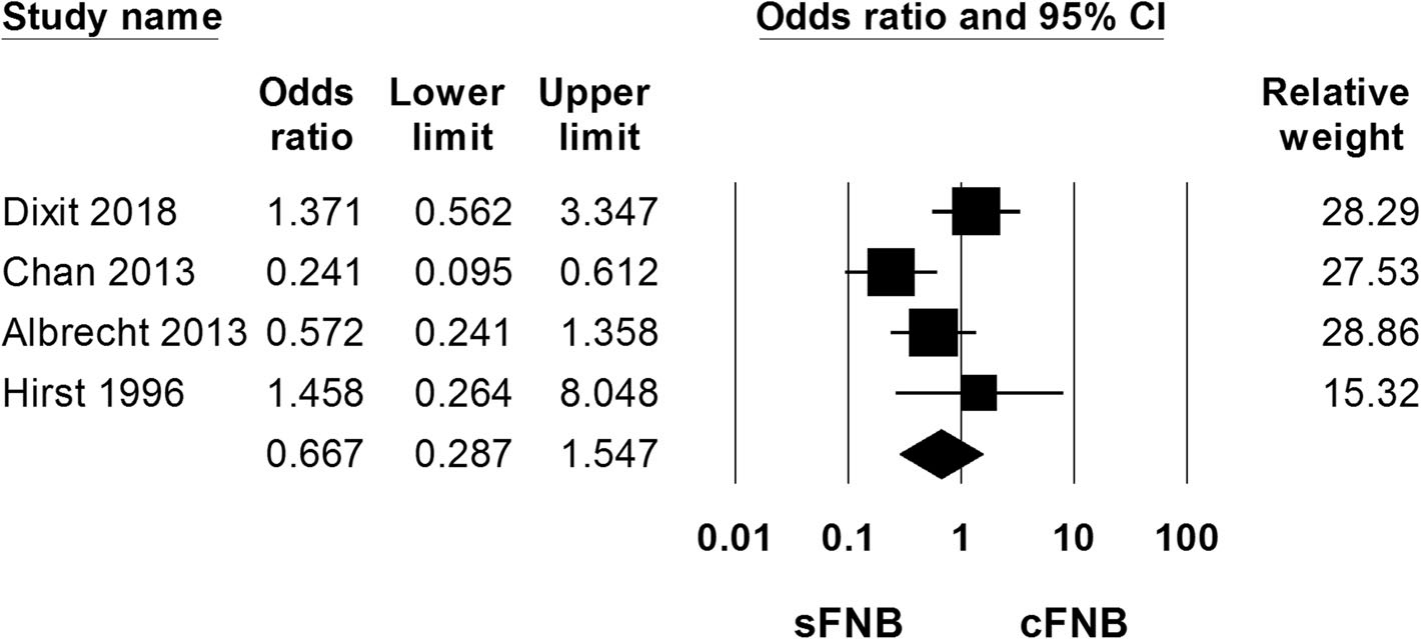
Comparison of continuous femoral nerve block (cFNB) versus single-injection femoral nerve block (sFNB) with regard to postoperative nausea rate

3 studies (334 patients) assessed postoperative nausea and the pooled data showed no significant difference between 2 group (OR: 0.667; 95% CI 0.287 to 1.547; Heterogeneity: I^2^ = 62.40; Fig. 7).

#### Risk of publication bias

**Fig. 8:**
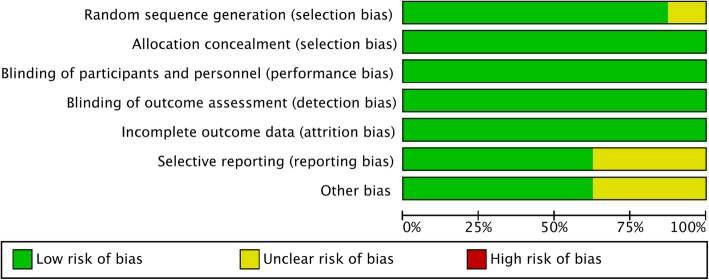
Assessment of the risk of bias

**Fig. 9:**
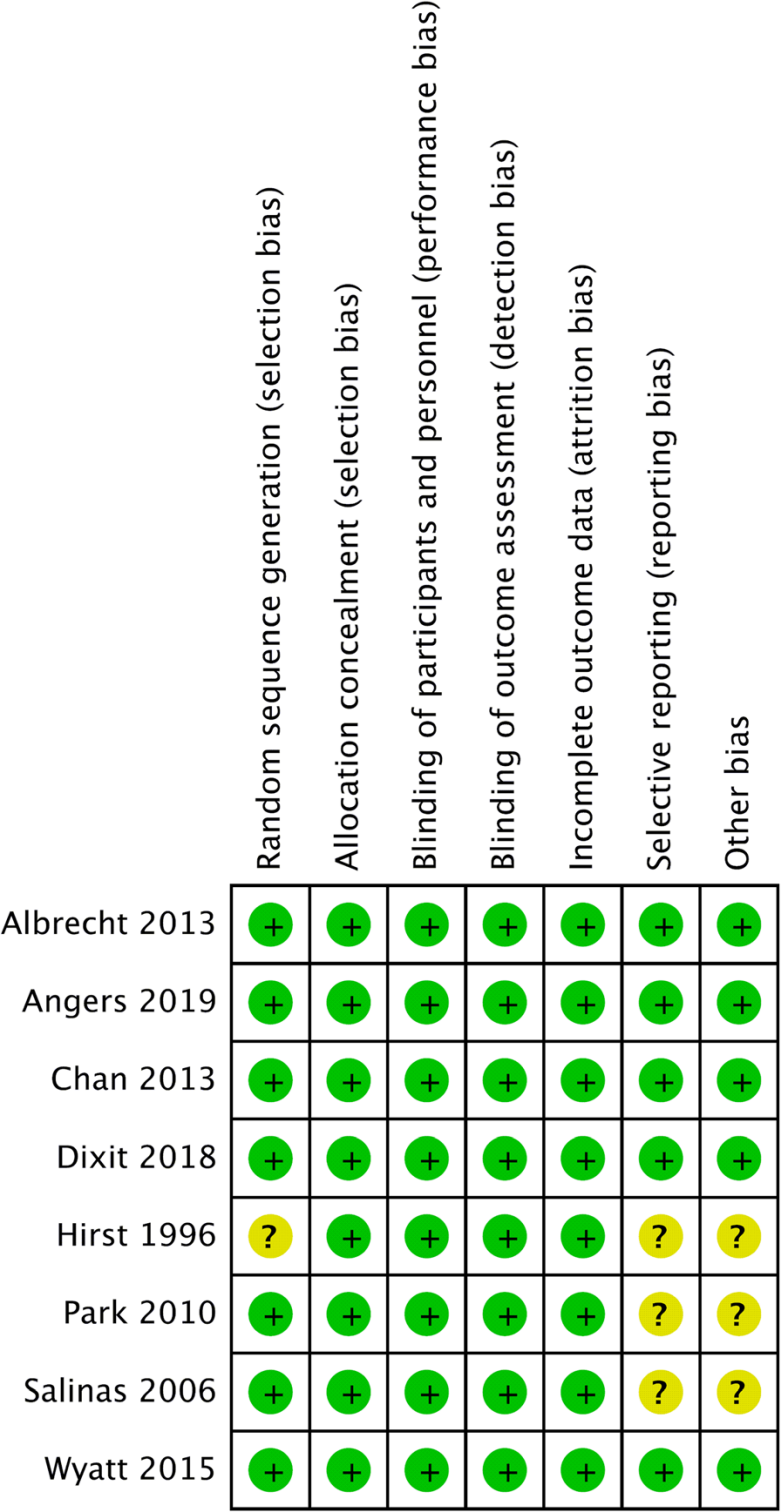
Results of risk of bias evaluation for the included study

The risk of publication bias is shown in Fig. 8 and Fig. 9. The random sequence generation (selection bias) was unclear in 1 of the 8 (12.5%) studies. The completeness of the reported data (reporting bias) was unclear in 3 of the 8 (37.5%) studies. In Fig. S1-S6, we demonstrated the funnel plots for SMD and log odds ratio of all the outcome domains from every study.

## Discussion

In this meta-analysis, we evaluated the efficacy of cFNB compared with that of sFNB in TKA. We included 8 studies with 626 patients. In comparison with the sFNB group, patients who received cFNB consumed less opioids at postoperative 24 and 48 h. VAS score at 24 h was lower in the cFNB group but did not meet the MCID for TKA [16]. Other outcome parameters including VAS scores at 48 h, length of hospital stay and postoperative nausea rates were not significant different.

Multimodal pain management in total joint arthroplasty involves a combination of modalities that act on different regions of the pain pathway to achieve better pain relief [2–4]. In the setting of multimodal analgesic protocols, a few studies reported a lower VAS score in the cFNB group [10, 11, 17], while other studies did not find a difference [9, 12, 18, 19]. Since many studies employed spinal anesthesia (or subarachnoid block), PCA and/or sciatic nerve block as part of their regimen, it could have greatly alleviated pain and may masked the efficacy of femoral nerve blocks. Therefore, it appears that there was no significant difference between the cFNB and sFNB group [9–12, 14, 17]. Results from this meta-analysis showed a lower VAS score at 24 h in the cFNB group compared with the sFNB group. However, based on the MCID for TKA determined by Danoff et al., this difference did meet the criteria for clinical significance [16]. Since sFNB was reported to have an analgesic effect up to 24 h [20, 21], cFNB was expected to extend the duration. However, the VAS score at 48 h was not different. The findings in pain scores is quite different from the results of meta-analysis conducted by Chan et al. in year 2014, in which the cFNB group was associated with a lower VAS score at both postoperative 24 and 48 h [13]. This might indicate that cFNB has limited add-on effect to sFNB with regards to pain scores in the setting of a multimodal analgesic protocol. In addition to assessing pain scores, consuming less amounts of opioids was another important issue that raises concerns [22–24]. We noted that patients can benefit from cFNB with regards to a reduced need of opioids in the early postoperative period (24 and 48 h), compared with the sFNB group.

Another important aim of a pain management modality is to facilitate rehabilitation and functional recovery. A variety of outcome parameters have been evaluated and compared between cFNB and sFNB with mixed results [12, 14, 17–19]. Chan et al. compared the time required to achieve several functional performance goals including range of motion to 90°, active straight leg raise, the need for ambulating assistance with walking aids and obstacle clearance between cFNB and sFNB [12]. The authors did not find a significant difference in either of the parameters and concluded that cFNB and sFNB were similar in facilitating postoperative rehabilitation. In addition, Albrecht et al. also noted similar results in early functional recovery (distance walked, active and passive knee flexion) as well as long-term patient reported outcomes (WOMAC score and SF-36 score) between the two groups [17]. Similarly, Wyatt et al. found no differences in knee range of movement on postoperative day 1, 2 and 3 [19]. Dixit et al. found a higher proportion of patients in the cFNB group were able to walk independently at their rehabilitation session [18]. In contrast to those studies listed above that reported little or no benefits from cFNB with regards to functional outcome, Angers et al. noted that both sFNB and cFNB were associated with a detrimental effect on short- to mid-term quadriceps strength, knee range of motion and WOMAC scores. Patients that received PCA alone performed better on these outcome domains compared with patients who received either cFNB or sFNB plus PCA. Interestingly, the effect of decreased quadriceps strength recovery in the cFNB and sFNB group could last up to 12 months [14]. We can only describe the functional outcomes qualitatively rather than to perform a quantitative analysis because of the heterogenous outcome domains used in the studies.

In the current study. cFNB did not lead to a shorter length of hospital stay compared with the sFNB group. Although cFNB was associated with less opioid consumption compared with the sFNB group, this benefit did not seem to translate to a faster functional recovery or a shorter length of hospital stay. In addition, the 5 studies that evaluated duration of hospital stay had different discharge criteria [10, 12, 14, 18, 19]. The heterogenous discharge criteria could greatly weaken the clinical implication of this parameter.

Postoperative fall has been one of the most common complications in patients receiving either cFNB or sFNB [25, 26]. Feibel et al. reported a large series of 1190 patients who underwent any form of knee arthroplasty and received cFNB for 2–3 days. Major falls and permanent femoral nerve injury were the most common complications [25]. Turbitt et al. reported a higher rate of falls from a large series of 3736 patients who had received cFNB [27]. As for sFNB, Sharma et al. evaluated 709 patients who received sFNB after TKA surgery. Falls were the most common complication and reoperations were required in 3 patients (0.4%). To compare the safety with regard to risk of fall between cPNB and sPNB, Ilfeld et al. found a higher rate of falls in patients who received a cPNB than patients with sPNB. This raises concerns for the safety of cFNB, especially in the setting of ERAS [28]. Wasserstein et al. evaluated the risk factors of inpatient falls after 2197 primary TKAs. The overall incidence of fall was 2.7%. Advanced age, BMI > 30 kg/m^2^ and cFNB were all independent risk factors that were associated with inpatient falls [29]. In our analysis, there were 346 patients included in the cFNB group and 280 in the sFNB group. Only 2 patients in the sFNB group had a fall. According to the reported incidence of fall [25–27], the relatively smaller sample size in this analysis might be insufficient to draw a conclusion on this rare but important adverse event. As stated by some studies, a comprehensive fall prevention care would be warranted to modify and lower the risk of falls after both sFNB and cFNB [12, 18, 30, 31].

There are some limitations that should be recognized. First, we searched only for English articles but not articles in other languages or unpublished data. This would be a potential source of publication bias. Second, there was a high heterogeneity between studies including age, gender, doses and regimens used in cFNB and sFNB, type of anesthesia and pain management modalities included in the multimodal analgesic protocol. Third, this meta-analysis compared the efficacy of cFNB and sFNB based on outcome domains including pain scores and opioid consumption. However, there is limited data in the studies so we could not validate whether these benefits can lead to enhanced recovery after surgery, improved long-term functional outcome and less total cost. In addition, the relatively small sample size made it difficult to compare the risk of several rare adverse events, especially falls related to the use of cFNB or sFNB. The incidence was evaluated in several case series with large sample size [25–27] but there is lack of RCTs to evaluate this outcome domain. Furthermore, it is interesting that Dixit et al. noted both cFNB and sFNB led to a decreased quadriceps strength up to 12 months, compared with the control group [18]. Some studies estimated that the return of quadriceps motor function or proprioception after discontinuation of cFNB were around 3 to 16 h [27, 32, 33]. Further studies should investigate the association of cFNB and sFNB postoperative quadriceps strength and to provide more information to formulate a fall prevention strategy.

## Conclusions

In the setting of a multimodal analgesic protocol, patients might benefit from a cFNB with regards to a reduced consumption of opioids in the early postoperative period. In terms of pain alleviation, both modalities provided substantial pain relief during the postoperative period and no clinically significant differences were noted between the two groups.

## Supplementary information

**Additional file 1.** Fig. S1. Funnel plot. (VAS score at postoperative 24 h).

**Additional file 2.** Fig. S2. Funnel plot. (VAS score at postoperative 48 h).

**Additional file 3.** Fig. S3. Funnel plot. (Total amount of opioids consumed at postoperative 24 h).

**Additional file 4.** Fig. S4. Funnel plot. (Total amount of opioids consumed at postoperative 48 h).

**Additional file 5.** Fig. S5. Funnel plot. (Length of hospital stay).

**Additional file 6.** Fig. S6. Funnel plot. (Postoperative nausea rate).

## Data Availability

The information to access the data used in the study is included within this article.

## Abbreviations

AOR: Adjusted odds ratio
CFNB: Continuous femoral nerve block
ERAS: Enhanced recovery after surgery
MCID: Minimal clinically important difference
PCA: Patient controlled analgesia
PRISMA: Preferred reporting items for systematic reviews and meta-analysis
RCTs: Randomized controlled trials
SFNB: Single-injection femoral nerve block
SMDs: Standardized mean differences
TKA: Total knee arthroplasty
VAS: Visual analog scale
